# Exploring the therapeutic synergy of drug-lifestyle interventions in fluorosis: a randomized trial on cardiovascular metabolic outcomes from the China fluorosis cohort (CFC)

**DOI:** 10.3389/fphar.2026.1737666

**Published:** 2026-03-31

**Authors:** Yun Lu, Fuyu Tao, Shaofeng Wei, Ting Hu, Rourou Wang, Chuan Ye, Feng Hong, Peng Luo

**Affiliations:** 1 Key Laboratory of Environmental Pollution Monitoring and Disease Control, Ministry of Education, School of Public Health, Guizhou Medical University, Guiyang, China; 2 Collaborative Innovation Center for Prevention and Control of Endemic and Ethnic Regional Diseases Co. Constructed by the Province and Ministry, Guizhou Medical University, Guiyang, China; 3 Guizhou Provincial Engineering Research Center of Ecological Food Innovation, Guizhou Medical University, Guiyang, China; 4 Department of Orthopedics, The Affiliated Hospital of Guizhou Medical University, Guiyang, China; 5 The State Key Laboratory of Functions and Applications of Medicinal Plants, School of Pharmaceutic Sciences, Guizhou Medical University, Guiyang, China

**Keywords:** cardiovascular metabolism, chronic fluorosis, efficacy evaluation, randomized intervention trial, traditional Chinese medicine (TCM) compound

## Abstract

**Introduction:**

Skeletal fluorosis patients face a markedly elevated risk of cardiovascular metabolic abnormalities, including endothelial dysfunction, excessive oxidative stress, and impaired calcium-phosphate metabolism. Current clinical interventions for this condition are limited to symptomatic analgesia and conventional anti-osteoporotic treatments, and lack targeted therapeutic strategies for the “kidney deficiency and blood stasis” pathogenesis of skeletal fluorosis that can synergistically protect both the skeletal and cardiovascular systems.

**Methods:**

This multicenter randomized controlled trial was conducted on 1,480 skeletal fluorosis patients from the China Fluorosis Cohort (CFC), to investigate the synergistic therapeutic effects of three traditional Chinese medicine (TCM) combinations—*Ginkgo biloba* combined with *Epimedium* (Drug 1), *Xianling Gubao* combined with *Eucommia ulmoides* (Drug 2), and *Gusongbao* combined with *Rosa roxburghii* (Drug 3)—in conjunction with lifestyle modifications on cardiovascular metabolic outcomes.

**Results:**

Smoking and alcohol consumption were identified as independent risk factors for reduced therapeutic efficacy in skeletal fluorosis patients with comorbid cardiovascular metabolic abnormalities (OR = 2.755, 95% CI: 1.400 –5.421). Among the three TCM combinations, Drug 2 and Drug 3 significantly counteracted these adverse risk factors, reducing the risk of treatment failure by 53.9% (OR = 0.461) and 57.0% (OR = 0.430), respectively. Drug 3 exhibited superior efficacy in reducing diastolic blood pressure (*β* = −2.263, *P* = 0.010), while its systolic blood pressure-lowering effect diminished with increasing age. Drug 2 showed synergistic benefits with improved sleep quality (*β* = 1.596, *P* = 0.002) and healthy dietary habits (*β* = −1.180, *P* = 0.001), which enhanced its antihypertensive effects and led to a significant reduction in low-density lipoprotein cholesterol (LDL-C) levels (*P* < 0.05). Additionally, the interaction models outperformed the main-effect models, confirming the dynamic synergistic effect between lifestyle interventions and TCM pharmacotherapy.

**Discussion:**

These findings highlight the clinical necessity of integrating behavioral interventions with pharmacotherapy to optimize cardiovascular metabolic safety in the clinical management of skeletal fluorosis. Based on these results, we propose a novel theoretical framework—Personalized Behavioral-Integrated Therapy (PBIT)—which guides the incorporation of patient-specific behavioral and demographic factors into tailored treatment strategies, thereby improving the precision and clinical outcomes of skeletal fluorosis treatment.

## Introduction

Chronic fluorosis is a systemic toxic disease caused by prolonged excessive fluoride intake, primarily through drinking water but also via air, food, tea, and other sources in specific natural environments. Its main clinical manifestations are dental fluorosis and skeletal fluorosis (Meena and Gupta, 2021). Millions of people worldwide are exposed to excessive fluoride via multiple pathways, primarily through contaminated drinking water, industrial pollution, and coal burning. It is endemic in at least 25 countries across the globe, with China and India being the worst-affected countries (Pramanik and Saha, 2017). Fluorosis in China is characterized by its wide distribution, complex disease zone types, large patient population, and significant challenges in diagnosing and treating health damage, making it a key focus for disease prevention and control (Zhao et al., 2011). The populations at risk from waterborne, coal-burning, and tea-drinking types of fluorosis in China are 72.07 million, 33.36 million, and 13.10 million, respectively (Sun et al., 2019). The policy focus has shifted from simple prevention measures to a comprehensive approach that combines medical treatment and prevention (Sun et al., 2023).

Traditional research on chronic fluorosis has long focused on skeletal system damage, primarily because bone is the main target organ for fluoride accumulation. After excessive fluoride ions are absorbed through the intestine, approximately 99% combine with hydroxyapatite and deposit in bones and teeth, directly causing abnormalities in bone mineralization and destruction of trabecular structure. This abnormal bone remodeling is a core trigger of bone pain: fluoride deposition forms fluoroapatite crystals that disrupt the dynamic balance of bone formation and resorption, leading to trabecular thickening, sclerosis, and uneven stress distribution in bone tissue, which stimulates nociceptive nerve endings in the periosteum and bone marrow cavity. Meanwhile, excess fluoride induces persistent oxidative stress and inflammatory responses in bone microenvironments, upregulating the expression of pro-inflammatory cytokines (e.g., TNF-α, IL-6) and reactive oxygen species (ROS), which further sensitize pain receptors and exacerbate inflammatory pain. In addition, fluoride-induced periosteal hyperplasia, ligament calcification, and joint capsule fibrosis cause mechanical compression of peripheral nerves and limit joint movement, resulting in secondary mechanical pain and activity-related discomfort. Manifested as typical symptoms of fluorosis such as neck and lower back joint pain and functional impairment. However, with the in-depth research in epidemiology and mechanisms, many studies have found that the toxic effects of fluoride exhibit characteristics of “multiple organs and multiple systems”: fluoride ions can be distributed throughout the body via the bloodstream, causing direct damage to bones and also leading to secondary damage to the cardiovascular metabolic system through mechanisms such as interfering with cellular metabolism, it can promote oxidative stress, inflammation, and endothelial dysfunction—key drivers of hypertension and atherosclerosis. Fluoride accumulates in aortic walls and is correlated with coronary calcification (Li et al., 2012). Electrocardiographic analyses of fluorosis patients revealed arrhythmia and reduced myocardial function (Zhou and Zhang, 1988; Xu and Xu, 1997). Mechanistic studies revealed that excess fluoride ions disrupt cardiovascular homeostasis via multiple pathways (Wölfl et al., 1996; Yang et al., 2024; Barbier et al., 2010). Serological analyses revealed elevated levels of inflammatory factors and lipid peroxidation products in fluoride-exposed populations (Sun et al., 2014). Clinical cohort studies have shown a higher incidence of hypertension and elevated cholesterol in chronically fluoride-exposed populations (Amini et al., 2011; Li et al., 2021; Varol and Varol, 2013).

Current therapeutic strategies—largely centered on single-drug interventions (e.g., traditional Chinese medicine, antioxidants) or isolated lifestyle recommendations. Even studies incorporating lifestyle interventions have predominantly focused on generalized lifestyle (Yang et al., 2017) recommendations (e.g., exercise promotion) rather than specific behavioral modifications (e.g., smoking cessation, dietary modification) known to interact with pharmacological mechanisms. For instance, a 2023 meta-analysis established that smoking exacerbates fluoride-induced oxidative stress by upregulating pro-inflammatory cytokines and impairing antioxidant defense systems in both animal models and human osteoblasts (Angwa et al., 2023). Similarly, alcohol consumption has been shown to alter lipid metabolism in fluoride-exposed populations; preclinical studies indicate that ethanol potentiates fluoride-induced vascular calcification and dyslipidemia through impairment of hepatic lipid transport and increased oxidative stress (Chauhan et al., 2013). Nevertheless, despite its documented potential to influence drug absorption and metabolic pathways, no clinical investigation has specifically examined alcohol as a modifier of therapeutic efficacy in fluorosis. Collectively, existing preclinical and clinical evidence provides a compelling rationale for developing integrated drug-lifestyle intervention strategies. In a 2022 cohort study, patients with skeletal fluorosis who received combined calcium supplementation with a low-fluoride diet presented a 37% greater reduction in bone pain than did those who received monotherapy (Angwa et al., 2023). Mechanistically, fluoride-induced disruption of lipid homeostasis (e.g., elevated LDL-C and triglyceride levels) may be mitigated by lifestyle modifications, which could potentiate drug effects (Xia et al., 2021). However, this synergy remains unexplored in fluorosis, with most trials evaluating drugs or behaviors in isolation (Pereira et al., 2016).

This study selected three Chinese patent medicine formulations—*Xianling Gubao Capsule*, *Ginkgo biloba Extract Tablets*, and *Gusongbao Granules*—along with three herbal materials: *Epimedium*, *Rosa roxburghi*i, and *Eucommia ulmoides*. In traditional Chinese medicine theory, *Xianling Gubao Capsule* functions to tonify the kidney and strengthen bone, activate blood circulation, and unblock collaterals. Collaterals are the fine, branching network of vessels that extend from the main meridians in the TCM meridian system. They permeate all tissues, organs, and bones of the body, acting as the primary pathway for transporting Qi (vital energy) and Blood to nourish every bodily structure. Unlike the main meridians (which carry Qi/Blood along major bodily pathways), collaterals ensure localized delivery of nutrients and remove metabolic waste at the tissue level. It is commonly used to treat osteoporosis, bone fractures, and soft tissue injuries (Bai et al., 2018). *Ginkgo biloba Extract Tablets* are known for promoting blood circulation, resolving stasis, unblocking collaterals, and alleviating pain. They have been traditionally applied in cases of chest impediment and heart pain, as well as wind-stroke and hemiplegia (Zhang et al., 2017). Wind-stroke is an acute TCM syndrome corresponding to cerebrovascular accidents (stroke) in Western medicine (including ischemic and hemorrhagic stroke). *Gusongbao Granules* act to tonify the kidney, activate blood, strengthen tendons, and reinforce bone. They are mainly indicated for bone wilting and bone pain caused by kidney deficiency and blood stasis (Lu et al., 2023). Among the three herbal materials, *Epimedium* is regarded as a key herb for warming kidney yang, strengthening bone and tendon, and dispelling wind-dampness (Chinese Pharmacopoeia Commission, 2020). *Eucommia ulmoides* is expert in tonifying the liver and kidney and strengthening bone and muscle, making it an essential herb for treating kidney deficiency-related lower back pain (Gao, 2007). *Rosa roxburghii* is known for strengthening the spleen, promoting digestion, clearing heat, and generating fluid. It is commonly used among Guizhou folk medicine for food accumulation, abdominal distension, summer heat, and thirst (Li and Ren, 2022). The traditional functions of these medications highly align with the pathogenesis of skeletal fluorosis, which is characterized by “kidney deficiency and blood stasis.” Kidney-tonifying herbs can replenish essence to strengthen bone, while blood-activating herbs resolve stasis to unblock collaterals and relieve pain. Modern pharmacological research has revealed the active components and mechanisms of these drugs. Icariin in *Xianling Gubao Capsule* promotes osteoblast differentiation and inhibits osteoclast activity via the Wnt/β-catenin signaling pathway (Zhang et al., 2016). *Ginkgo* flavonoid glycosides and terpene lactones in *Ginkgo biloba* Extract exhibit strong antioxidant activity, scavenging free radicals and improving microcirculation through inhibition of platelet-activating factor (Liu et al., 2015). Active components in *Gusongbao Granules* significantly increase bone density and improve bone biomechanical properties, mechanisms related to the regulation of bone metabolism signaling pathways (Lu et al., 2023). Among the herbal materials, icariin is considered a key component promoting bone formation (Zhang et al., 2008). Geniposide and aucubin in *Eucommia ulmoides* facilitate collagen synthesis and exert cartilage-protective effects (He et al., 2014). *Rosa roxburghii* is rich in vitamin C and superoxide dismutase, providing potent antioxidant defense, while its chlorogenic acid contributes anti-inflammatory activity (Fu et al., 2020). Through multi-target and multi-pathway synergistic effects, these active components collectively exert antioxidant, anti-inflammatory, bone metabolism regulatory, and microcirculation-improving pharmacological actions, providing a scientific basis for alleviating skeletal degeneration and dysfunction associated with skeletal fluorosis.

Therefore, using data from the China Fluorosis Cohort Study (CFC), we assessed the efficacy of drug-lifestyle synergistic interventions on cardiovascular-metabolic outcomes with three traditional Chinese medicine compound formulations: *Ginkgo biloba* and *Epimedium*, *Xianling Gubao capsules* and *Eucommia ulmoides*, and *Gusongbao granules* and *Rosa roxburghii*. This approach addresses two critical research voids: (1) therapeutic synergy, which involves quantifying how specific lifestyle factors (e.g., smoking status and dietary fat intake) affect the efficacy of drugs on skeletal lesions and cardiovascular markers (blood pressure and lipid profiles); and (2) metabolic safety, which involves evaluating whether combined interventions reduce cardiovascular risk better than monotherapy does, particularly in elderly patients with comorbid hypertension (Li et al., 2021; Rejnmark et al., 2010; Spence and Weaver, 2013) and providing precise medication references for patients with fluorosis of the bone complicated by cardiovascular diseases.

## Materials and methods

### Participants

This study, funded by the National Key Research and Development Program of China in 2022, “Key Technologies for Early Detection and Precision Diagnosis and Treatment of Endemic Fluorosis” (Project No. 2022YFC2503003), focuses on patients with skeletal fluorosis aged 40 years and above who voluntarily participated in the cohort study. The inclusion criteria are strictly in line with professional standards: first, patients with a long-term living history in endemic fluorosis areas and diagnosed with endemic skeletal fluorosis according to WS/T 192–2021 “Diagnostic Criteria for Endemic Skeletal Fluorosis”; second, individuals who voluntarily participate and sign the informed consent form. With respect to the exclusion criteria, individuals with abnormal language expression or comprehension abilities, as well as those who took antihypertensive or lipid-regulating medications before and during the intervention, were not included in the study, ensuring the accuracy and validity of the research data.

### Study design

This randomized controlled trial (RCT) enrolled 1802 skeletal fluorosis patients, utilizing village-based grouping for concealed allocation (the same village received identical treatments: Drug 1 (*Ginkgo biloba* + *Epimedium*), Drug 2 (*Xianling Gubao capsules* + *Eucommia ulmoides*), and Drug 3 (*Gusongbao granules* + *Rosa roxburghii juice*). A partially blinded design was implemented: site physicians/pharmacists (unblinded for drug dispensing/guidance) and telephone adherence staff (unblinded for dose ratio checks) knew treatment assignments, whereas BP measurers (standardized protocol, blinded to groups) and lipid analysis teams (central laboratory with coded samples, blinded data management) remained blinded. After 3 months of treatment, 322 patients were excluded (adverse events, BP/lipid-affecting drugs), leaving 493–495 patients per group. Adherence monitoring included bi-weekly follow-ups (assessing dose ratios, interventions for non-adherence).

The primary outcome indicators include ordinal categorical variables (efficacy grading of skeletal fluorosis severity: no therapeutic effect, therapeutic effect, and marked therapeutic effect) and continuous variables (changes in blood pressure and lipid indices). Skeletal fluorosis is graded into three grades: Grade I (symptoms, no signs), Grade II (typical manifestations, can do some labor), and Grade III (lost work ability). Covariate analysis involves treatment groups, lifestyle variables, and confounding factors such as sex, age, and baseline blood pressure and lipid conditions. Pain intensity was assessed using the visual analog scale (VAS). This tool is characterized by simplicity in operation and high sensitivity and is suitable for quantifying the subjective pain level of most conscious patients. A score of ≤3 indicates mild pain, a score of 4–6 indicates moderate pain, and a score of ≥7 indicates severe pain.

**FIGURE 1:**
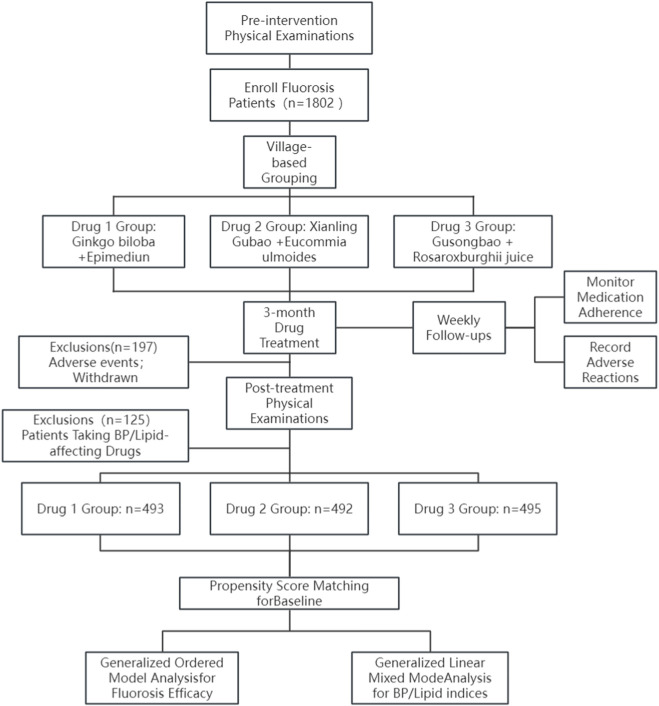
Experimental design process.

This design ensured scientific rigor by addressing multicenter village-level baseline imbalances through statistical matching, combining concealed allocation with partial blinding to balance practicality (drug delivery/monitoring) and bias reduction (outcome measurement/analysis), leveraging appropriate models for ordinal/continuous data while controlling for confounding factors (Figure 1).

The study successfully passed the review of the Human Experiment Ethics Committee of Guizhou Medical University (Ethics Approval No. 2023 Review No. 189), which strictly adhered to ethical norms. Moreover, the project has been registered with the Chinese Clinical Trial Registry (Registration No. ChiCTR2300075706; Registration Date: September 13, 2023; Registration Website: http://www.chictr.org/cn/), ensuring the scientific nature and standardization of the study.

Life behavior score

Life behaviors were categorized into four domains with standardized scoring:

1. Smoking and drinking (0–11 points; higher = greater severity),
2. Sleep quality (0–7 points; higher = poorer quality),
3. Dietary Patterns (3–16 points; higher = better balance),
4. Physical activity (0–3 points; higher = greater activity).

The detailed scoring criteria for each domain are provided in Supplementary Appendix Table 1.

The sample size for this randomized controlled trial was determined *a priori* using G*Power software (version 3.1.9.7). Based on previous studies investigating TCM interventions in skeletal fluorosis, we assumed a medium effect size (Cohen’s *f* = 0.25) for the primary outcome (change in skeletal fluorosis grading). With an alpha level of 0.05 and a power of 0.90, the required sample size for a one-way ANOVA with three groups was approximately 1,200 participants. Accounting for an anticipated dropout rate of 20%, we aimed to recruit at least 1,500 participants. The final enrolled sample of 1,802 participants exceeded this requirement, ensuring adequate statistical power to detect clinically meaningful differences in both primary and secondary outcomes.

### TCM interventions

To identify effective therapeutic agents for skeletal fluorosis, this study first conducted a literature review to initially determine three compound formulas (*Ginkgo biloba*, *Gu Song Bao Granules*, *Xianling Gubao Capsule*) and three medicinal adjuvants (*Epimedium*, *Rosa roxburghii*, *Eucommia*) that have the potential to ameliorate the symptoms and signs of skeletal fluorosis. Subsequently, network pharmacology was employed: chemical components and their corresponding SMILES numbers of the screened drugs were retrieved from the TCM and Chemical Component Database (http://www.organchem.csdb.cn/scdb/main/tcm_introduce.asp), HERB (http://herb.ac.cn/) and literature, with unrecorded components supplemented by PubChem (https://pubchem.ncbi.nlm.nih.gov/) for SMILES numbers; after merging and deduplication, candidate active components were screened via SwissADME (http://www.swissadme.ch/) with the criteria of high gastrointestinal absorption (GI) and at least three positive results for drug likeness. Candidate active component targets were predicted by SwissTargetPrediction (http://swisstargetprediction.ch/), and duplicate targets were removed and protein names standardized via the UniProt database (https://www.uniprot.org/). For disease target collection, fluorosis-related targets were retrieved from GeneCards (https://www.genecards.org/) using the keywords “fluorosis” and “endemic fluorosis”, with duplicate targets deleted and protein names also corrected by UniProt (https://www.uniprot.org/) to establish a disease target dataset. Potential targets of traditional Chinese medicines and fluorosis were identified by intersecting drug component targets with disease targets via Venn diagrams generated by Venny 2.1.0 (https://bioinfogp.cnb.csic.es/tools/venny/), and a protein-protein interaction (PPI) network was constructed by importing these potential targets into STRING 11.5 (https://string-db.org/) (*Homo sapiens*, medium confidence >0.4). Furthermore, Gene Ontology (GO) functional enrichment analysis and Kyoto Encyclopedia of Genes and Genomes (KEGG) pathway enrichment analysis were performed on the disease-related network via Metascape (https://metascape.org/gp/index.html#/main/step1) to clarify the core targets for drug screening. Based on the “disease-compound-target” network and the principle that “potential targets of the prescription drugs should maximize coverage of the core disease targets”, three potential intervention formulas for skeletal fluorosis were screened out, namely, *Ginkgo biloba* combined with *Epimedium*, *Xianling Gubao Capsule* combined with *Eucommia*, and *GuSongBao Granules* combined with *Rosa roxburghii*. Finally, the network prediction results were validated through virtual screening, *in vitro* experiments, and *in vivo* verification, and the above three formulas were ultimately confirmed as candidate drug regimens for skeletal fluorosis intervention research.

*Xianling Gubao Capsule*, *Ginkgo biloba Tablets*, and *Gu Song Bao Granules* are commercially available finished pharmaceutical products in capsule or granule form, procured directly from manufacturers. For *Epimedium* and *Eucommia*, processed raw materials were employed, whereas a concentrated juice formulation was used for *Rosa roxburghii*. The dosage of all administered agents was determined in accordance with the manufacturers' prescribing guidelines.

### Statistical analyses

The normality of the data was assessed using the Shapiro-Wilk test. Quantitative data with a normal distribution are presented as the mean ± standard deviation, whereas skewed data are expressed as the median (interquartile range, IQR). Categorical data are presented as frequencies (n) and percentages (%). The Kruskal–Wallis H test was used to compare differences in quantitative data, and the chi-square test was applied for categorical variables.

To balance confounders effectively, individual matching was performed across the three medication groups based on propensity scores, using 1:1 non-replacement nearest neighbor matching with a caliper parameter set at 0.3 to construct baseline-balanced subsamples. Standardized weights were then calculated to align the distributions of age and baseline blood pressure/lipid levels across groups, ensuring comparability.

For efficacy analysis of skeletal fluorosis severity grading, a generalized ordered logit model was employed, which relaxes the proportional odds assumption and allows covariate effects to differ across cutoffs. The model evaluated the main effects and interactions of treatment groups and life behavior scores, with the specific formula as follows:

$$\log\left(\frac{\text{Cumulative}\;\text{Probability}\;\left(\text{Grade}\leq j\right)}{\text{Cumulative}\;\text{Probability}\;\left(\text{Grade}>j\right)}\right)=\beta_{0j}+\beta_{1}\text{Drug}+\beta_{2}\text{Age}+\sum_{i=1}^{4}\beta_{i+2}{\text{Behavior}\;\text{Score}}_{i}+\text{InteractionTerms}$$

For continuous variables (blood pressure and lipid levels), a linear mixed effects model was applied. Given the non-normal distribution of data, a gamma distribution with a log link function was specified. Model parameters were fitted using the restricted maximum likelihood (REML) method to evaluate treatment groups, life behavior scores, and their interactions while adjusting for life behavior scores and age. The model formula is as follows:

$$yij=\beta_{0}+\beta_{1}\text{Dru}g_{i}+\beta_{2}\text{Ag}e_{i}+\sum_{k=1}^{4}\beta_{2+k}{\text{Behavior}\;\text{Score}}_{ik}+\text{InteractionTerm}s_{ij}+b_{i}+\in_{ij}$$

All the models included:

1. Main effects: Treatment groups and lifestyle factors;
2. Interactions: Treatment group × lifestyle factor (to assess how different lifestyles modify treatment efficacy).

For hypothesis testing and model diagnostics, the generalized ordered logit model evaluates the goodness-of-fit by comparing the current model with interaction effects against an intercept-only null model to assess the model’s explanatory power for the data. Conversely, the linear mixed effects model quantifies the contribution of interaction terms to model explanatory power through the difference in *R* ^*2*^ values between the model with main effects only and the model incorporating interaction effects, thus scientifically evaluating how well the model fits the sample data.

All analyses were conducted using R software version 4.4.3, with the significance level set at α = 0.05 (two-tailed test). Effect sizes will be reported as odds ratios (OR) with 95% confidence intervals or regression coefficients (*β*) with standard errors (SE).

## Results

### Study participants

**FIGURE 2:**
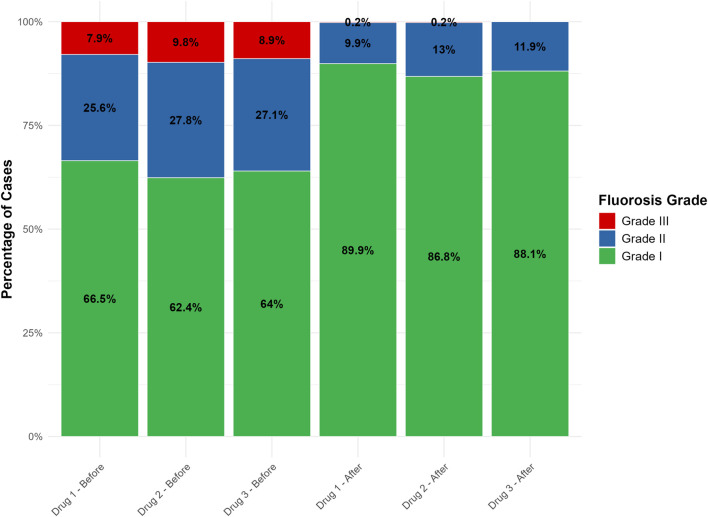
Generalized ordered logit model forest plot of skeletal fluorosis grading effects.

Baseline analysis revealed no significant difference in the degree of improvement in skeletal fluorosis severity among the three groups. The age distribution differed significantly (Kruskal–Wallis, *H* = 14.664, *P* = 0.00065) and was adjusted as a covariate. The sex distribution was similar (χ^2^ = 0.69072, *P* = 0.708). Intergroup differences existed in baseline systolic blood pressure (*H* = 8.712, *P* = 0.013) and total cholesterol (*H* = 7.540, *P* = 0.023) but not in diastolic blood pressure, triglycerides, LDL, or HDL. Propensity score matching balanced age, systolic blood pressure, and total cholesterol. Continuous variables were moderately correlated with systolic/diastolic blood pressure, total cholesterol/LDL, and HDL/LDL changes; weakly negatively correlated with HDL-TG levels; and weakly correlated with lifestyle indicators. The distributions are shown in Table 1 and Figure 2.

**TABLE 1:** Baseline characteristics of the three treatment groups.

Variables	Drug 1	Drug 2	Drug 3	Statistic	*P*. Value
Fluorosis grade, n (%)					
I	328 (66.5%)	307 (62.4%)	317 (64.0%)	2.109	0.716
II	126 (25.6%)	137 (27.8%)	134 (27.1%)		
III	39 (7.9%)	48 (9.8%)	44 (8.9%)		
VAS score	​	​	​	5.011	0.082
Gender, n (%)					
Female	295 (59.8%)	301 (34.0%)	290 (58.6%)	0.691	0.708
Male	198 (40.2%)	191 (38.8%)	205 (41.4%)		
Age, M (IQR)	61 (15)	64 (16)	62 (15.5)	14.664	0.001
Efficacy of skeletal fluorosis grading, n (%)					
No therapeutic effect	355 (72.0%)	348 (70.7%)	361 (72.9%)	0.221	0.900
Therapeutic effect	99 (20.1%)	97 (19.7%)	90 (18.2%)		
Marked therapeutic effect	39 (7.9%)	47 (9.6%)	44 (8.9%)		
Blood pressure, M (IQR)					
DBP	83 (18)	84 (18)	83 (18)	5.248	0.073
SBP	137 (29)	143 (28)	139 (28)	8.712	0.013
Blood lipids, M (IQR)					
HDL	1.42 (0.57)	1.45 (0.61)	1.42 (0.49)	2.527	0.283
LDL	2.87 (1.11)	2.83 (1.35)	2.81 (1.09)	1.642	0.440
TC	5.01 (1.44)	5.19 (1.60)	5.14 (1.38)	7.540	0.023
TG	1.19 (0.96)	1.32 (1.05)	1.28 (0.95)	2.636	0.268

VAS (Visual Analog Scale); DBP (Diastolic Blood Pressure); SBP (Systolic Blood Pressure); HDL (High-Density Lipoprotein); LDL (Low-Density Lipoprotein); TC (Total Cholesterol); TG (Triglycerides); M (Median); IQR (Interquartile Range).

### Propensity score matching (PSM)

**FIGURE 3:**
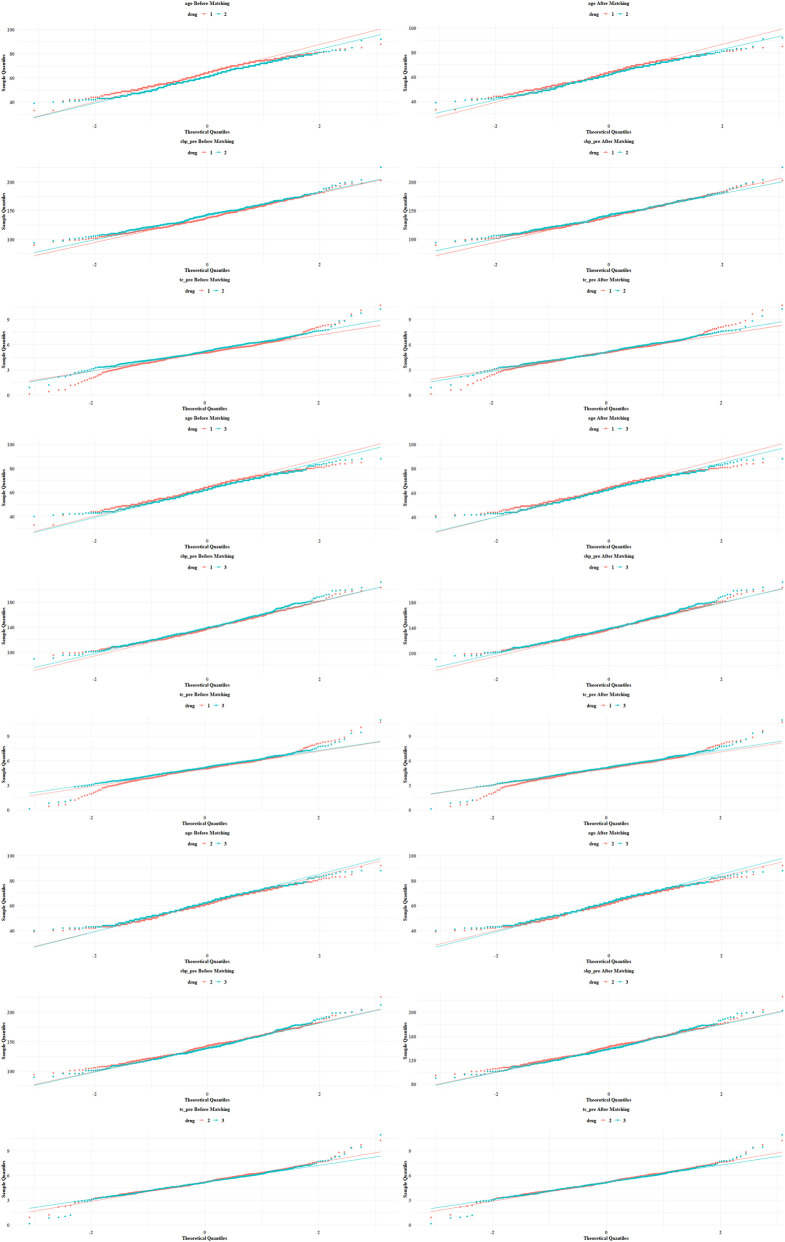
Visualization of Drug-behavioral determinant interactions.

After matching, the number of cases in each group was 449 for Drug 1 and Drug 2, 458 for Drug 2 and Drug 3, and 457 for Drug 1 and Drug 3. The standardized mean difference (SMD) of all variables was <0.1, indicating an excellent matching effect with no obvious unbalanced variables. Clinical indicators such as systolic blood pressure, diastolic blood pressure, and blood lipids were well balanced, making them suitable for subsequent intergroup comparisons. The distributions before and after propensity score matching are shown in Figure 3. The statistical power analysis demonstrated that for blood pressure and blood lipids, the current sample size achieved a detection power exceeding 99% (Group 12: 99.4%, Group 23: 99.7%, Group 13: 99.6%) for medium effect sizes (Cohen’s d = 0.3) at a significance level of *α* = 0.05. For skeletal fluorosis grading, the power remained >99.999%, even for small effect sizes (*f* ^*2*^ = 0.05). These results indicate that the study design possesses robust statistical power.

### Efficacy of skeletal fluorosis

A generalized ordered logit regression analysis was performed on the efficacy of skeletal fluorosis grading before and after the intervention to explore the differences in efficacy among different behavioral habits. The model converged after 5 iterations, and no Hauck-Donner effect was found (indicating stable parameter estimation). The results revealed that there were no significant differences in the efficacy of the three different treatment groups for skeletal fluorosis grading.

**FIGURE 4:**
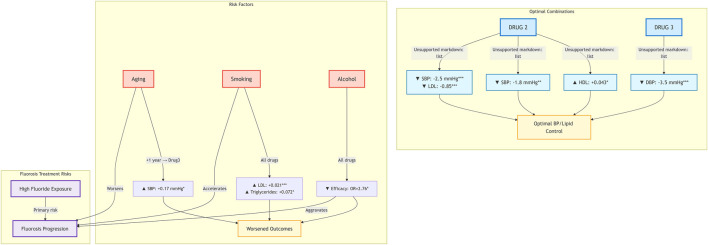
Distribution of skeletal fluorosis before and after intervention.

The generalized ordered logit model results revealed that smoking and drinking were independent risk factors (OR = 2.755, 95% CI: 1.400–5.421), whereas the main effects of age, gender, sleep quality, dietary habits, and physical activity were not significant. The univariate effects of Drug 2 and Drug 3 were not significant (OR = 7.968 and 10.814, 95% CI including 1), but their interactions with smoking and drinking were significant. Compared with Drug 1, Drug 2 + smoking/drinking and Drug 3 + smoking/drinking reduced the outcome risk by 53.9% (OR = 0.461, 95% CI: 0.222–0.958) and 57.0% (OR = 0.430, 95% CI: 0.205–0.903), respectively, compared with Drug 1, suggesting better efficacy in smokers/drinkers. The interaction effects of other drug-behavioral factors were not significant. The results are shown in Table 2 and Figure 4.

**TABLE 2:** Generalized ordered logit model results for efficacy of skeletal fluorosis grading.

Factor	OR	95% CI	*P*
Fixed effects			
Age	1.017	0.991–1.043	0.196
Gender	1.320	0.815–2.138	0.259
Behavioral factors			
Smoking and drinking	2.755	1.400–5.421	0.033
Sleep quality	1.229	0.881–1.716	0.225
Dietary habits	1.144	0.935–1.400	0.191
Physical activity	1.120	0.720–1.743	0.615
Drug comparison (reference: Drug 1)			
Drug2	7.968	0.169–35.233	0.725
Drug3	10.814	0.367–38.412	0.783
Drug-behavior interaction (reference: Drug 1)			
Drug 2 + smoking and drinking	0.461	0.222–0.958	0.038
Drug 3 + smoking and drinking	0.430	0.205–0.903	0.026
Drug 2 + sleep quality	0.943	0.605–1.469	0.251
Drug 3 + sleep quality	0.934	0.605–1.441	0.219
Drug 2 + dietary habits	0.843	0.641–1.107	0.220
Drug 3 + dietary habits	0.811	0.632–1.042	0.101
Drug 2 + physical activity	0.953	0.504–1.805	0.701
Drug 3 + physical activity	1.043	0.605–1.798	0.682

Goodness-of-fit: Resid. df decreased from 1460 (Model 1) to 1450 (Model 2), a reduction of 10 df due to 10 explanatory variables. Resid. Dev decreased from 1084.94 to 933.55, indicating better fit for the full model. LR, stat. was 151.39 (P < 0.001), confirming significant fit difference between full and null models.

OR (Odds Ratio); CI (Confidence Interval).

Post-intervention, all the groups presented significantly reduced VAS pain scores. Drug main effects indicated that Drug 3 significantly lowered the VAS score compared with Drug 2 (*β* = −1.180, SE = 0.575, p = 0.040), with no significant differences between Drug 1 and Drug 2 (*β* = 0.419, SE = 0.581, *P* = 0.471) or Drug 1 and Drug 3 (*β* = −0.645, SE = 0.529, *P* = 0.223). For behavioral habits, improved diet had the strongest analgesic effect across all drug comparisons (all *P* < 0.001), whereas poor sleep correlated with increased pain (*P* = 0.024 to <0.001). Significant drug-behavior interactions were observed: Drug 2 weakened exercise analgesia (*β* = −0.193, *P* = 0.030), and Drug 3 reduced dietary benefits (*β* = 0.078, *P* = 0.044). Female gender and older age were correlated with greater pain (all *P* < 0.001). The results are shown in Table 3.

**TABLE 3:** Generalized linear mixed model results for the VAS score.

Factors	Drug 1 vs 2			Drug 1 vs 3			Drug 2 vs 3		
	*β*	*SE*	*p*	*β*	*SE*	*p*	*β*	*SE*	*p*
Intercep-t1	0.378	0.531	0.477	0.687	0.513	0.180	0.479	0.577	0.407
Demographics									
Age	0.055	0.005	0.001	0.053	0.005	0.001	0.060	0.004	0.001
Gender	0.616	0.098	0.001	0.548	0.095	0.001	0.655	0.095	0.001
Main effect									
Drug	0.419	0.581	0.471	−0.645	0.529	0.223	−1.180	0.575	0.040
Smokingand drinking	0.081	0.051	0.111	0.072	0.047	0.123	0.134	0.043	0.002
Sleep quality	0.109	0.048	0.024	0.113	0.047	0.016	0.224	0.047	0.001
Dietary habits	−0.149	0.029	0.001	−0.146	0.029	0.001	−0.152	0.029	0.001
Physical activity	0.128	0.058	0.028	0.091	0.056	0.105	−0.081	0.064	0.205
Interaction effect									
Drug+ Smoking and drinking	0.059	0.066	0.369	0.087	0.061	0.158	0.020	0.058	0.726
Drug+ Sleep quality	0.104	0.069	0.131	0.055	0.065	0.398	−0.061	0.065	0.351
Drug+ Dietary habits	0.001	0.041	0.981	0.059	0.039	0.131	0.078	0.039	0.044
Drug+ Physical activity	−0.193	0.088	0.030	−0.007	0.081	0.930	0.146	0.085	0.087

R^*2*^ for main effect = 0.524.

R^*2*^ for interaction effect = 0.698.

VAS (Visual Analog Scale); β (regression coefficient); SE (standard error).

### Efficacy of blood pressure and lipids

The results revealed no significant differences in baseline systolic blood pressure among the three drug groups (all P > 0.05). Age had heterogeneous effects on drug efficacy, with each additional year associated with a 0.170 mmHg greater increase in systolic blood pressure in the Drug 3 group than in the Drug 1 group (*P* = 0.014), whereas sex had no significant influence (all *P* > 0.05). Although the antihypertensive effects of Drug 3 tended to be superior to those of Drug 1 (*β* = −1.726, *P* = 0.198), this difference did not reach statistical significance. Lifestyle factors significantly modulated treatment outcomes, with smoking and alcohol consumption attenuating drug efficacy (Drug 3 vs. Drug 1: *P* = 0.002; Drug 3 vs. Drug 2: *P* = 0.021), whereas improved sleep quality (*β* = 1.596, *P* = 0.002) and healthier dietary habits (*β* = −1.180, *P* = 0.001) enhanced the antihypertensive effects of Drug 2. Interaction analysis revealed synergistic effects between drug treatments and lifestyle modifications (Drug 2 vs. Drug 3 for sleep quality: *β* = −2.267, *P* = 0.018), with the interaction model (R^2^ = 0.620) demonstrating greater explanatory power than the main effects model alone (R^2^ = 0.489).

For diastolic blood pressure, age had a consistent positive influence across all drug comparisons (Drug 1 vs. Drug 2: *β* = 0.118, *P* = 0.017; Drug 1 vs. Drug 3: *β* = 0.094, *P* = 0.038), whereas female sex was associated with greater diastolic blood pressure increases specifically in the Drug 1 versus Drug 3 comparison (*β* = 2.117, *P* = 0.025). Compared with Drug 1, Drug 3 significantly improved diastolic blood pressure reduction (*β* = −2.263, *P* = 0.010), with a similar trend observed between Drug 2 and Drug 3 (*β* = −1.574, *P* = 0.090). Smoking and alcohol consumption universally impaired diastolic blood pressure control (all *P* < 0.05), whereas dietary improvements (Drug 1 vs. Drug 2: *β* = −0.666, *P* = 0.002; Drug 2 vs. Drug 3: *β* = −0.848, *P* = 0.001) and enhanced sleep quality (Drug 1 vs. Drug 2: *β* = 0.758, *P* = 0.030) selectively improved outcomes. Significant drug-lifestyle interactions were observed, particularly for sleep quality (Drug 2 vs. Drug 3: *β* = −1.472, *P* = 0.028) and dietary habits (Drug 1 vs. Drug 2: *β* = −1.032, *P* = 0.012), which augmented the effects of Drug 2, whereas physical activity appeared to enhance the efficacy of Drug 3 (Drug 1 vs. Drug 3: *β* = 2.201, *P* = 0.006; Drug 2 vs. Drug 3: *β* = 2.335, *P* = 0.008).

Analysis of lipid profiles revealed that age (Drug 1 vs. Drug 2: *β* = 0.021, *P* < 0.001; Drug 1 vs. Drug 3: *β* = 0.011, *P* < 0.001; Drug 2 vs. Drug 3: *β* = 0.014, *P* < 0.001) and female sex (all *P* < 0.01) were associated with elevated LDL levels across all drug groups, whereas healthy dietary habits significantly reduced LDL, particularly in the Drug 1 versus Drug 2 and Drug 2 versus Drug 3 comparisons (*P* < 0.05). Drug type did not significantly influence LDL changes (all *P* > 0.05), although smoking and alcohol consumption consistently increased LDL levels (*P* < 0.05). Compared with Drug 1, Drug 3 was associated with lower HDL levels (*β* = −0.066, *P* = 0.040), whereas improved dietary habits (Drug 2 vs. Drug 3: *β* = −0.043, *P* = 0.004) and physical activity (*P* < 0.05) increased HDL levels, particularly with Drug 2. Total cholesterol reduction was more pronounced with Drug 2 than with Drug 1 (*β* = −0.228, *P* = 0.024), although smoking and alcohol consumption attenuated this effect (Drug 2 vs. Drug 3: *β* = 0.086, *P* = 0.003; Drug 1 vs. Drug 2 interaction: *β* = 0.151, *P* = 0.039). Triglyceride levels were positively associated with age (Drug 1 vs. Drug 2: *β* = 0.008, *P* = 0.041; Drug 1 vs. Drug 3: *β* = 0.010, *P* = 0.003), whereas smoking increased triglycerides specifically in the Drug 1 versus Drug 2 comparison (*β* = 0.072, *P* = 0.006), with no significant drug-specific effects observed (*P* > 0.05). The specific results can be found in Supplementary Appendix Table 2.

## Discussion

This study, utilizing data from the China Fluorosis Cohort (CFC), provides the first systematic evidence supporting the benefits of combined pharmacological and lifestyle interventions in fluorosis management. The generalized ordered logit model identified smoking and alcohol consumption as independent risk factors for cardiovascular-metabolic abnormalities (OR = 2.755, 95% CI: 1.400–5.421). Notably, this risk was significantly attenuated through interactions with Drug 2 (*Xianling Gubao capsules* + *Eucommia ulmoides*) and Drug 3 (*Gusongbao granules* + *Rosa roxburghii*), which reduced cardiovascular-metabolic risk by 53.9% (OR = 0.461) and 57.0% (OR = 0.430), respectively. Mechanistically, the detrimental effects of smoking and alcohol may stem from their interference with fluoride metabolism and exacerbation of oxidative stress (Johnston and Strobel, 2020; Li et al., 2018; Sheikh and Collaborators, 2018). Smoking increases polycyclic aromatic hydrocarbons, which induce liver enzymes and accelerate drug metabolism, thereby reducing treatment efficacy (Kroon, 2007; Khudhur et al., 2025). In fluorosis, smoking may enhance fluoride-induced oxidative stress by elevating NADPH oxidase activity and reactive oxygen species (ROS) production, disrupting bone cell metabolism. (Loffredo et al., 2018). Alcohol consumption impairs calcium absorption and metabolism (Johnson et al., 2022), and ethanol has been shown to inhibit osteoblast activity while promoting osteoclast-mediated bone resorption, synergizing with fluoride-induced bone toxicity (Nyquist et al., 2002). In terms of blood pressure management, Drug 3 resulted in a more pronounced reduction in diastolic blood pressure compared to Drug 1 (*β* = −2.263, *P* = 0.010), and its synergistic effect with exercise was substantially enhanced (*β* = 2.335, *P* = 0.008). Drug 2 demonstrated significant synergistic effects with improved sleep (*β* = −1.596, *P* = 0.002) and a healthy diet (*β* = −1.180, *P* = 0.001), improving systolic blood pressure control. Regarding lipid metabolism, Drug 2 combined with a healthy diet showed synergistic benefits in reducing total cholesterol (TC) and low-density lipoprotein (LDL) (*β* = −0.228, *P* = 0.024). However, the effect of Drug 3 on high-density lipoprotein (HDL) warrants caution from a metabolic safety perspective.

Drugs 2 (*Xianling Guobao capsules* + *Eucommia ulmoides*) and 3 (*Gusongbao granules* + *Rosa roxburghii juice*) can counteract the adverse effects of smoking/alcohol consumption, potentially through their antioxidant and bone metabolism-regulating properties (Yang et al., 2022). The icariin in *Xianling Gubao* has antioxidant and anti-inflammatory effects, inhibiting the release of inflammatory factors such as TNF-α and IL-6, thereby alleviating fluoride-induced inflammatory responses in bone tissue (Yao et al., 2017). The *Eucommia* polysaccharides and chlorogenic acid in *Eucommia* promote osteoblast proliferation, inhibit osteoclast activity, and regulate bone metabolic balance. The ingredients in *Gusongbao Granules*, such as *Epimedium*, also have kidney-tonifying and bone-strengthening effects (Kwak et al., 2013; Shen et al., 2024). The vitamin C and superoxide dismutase (SOD) activities in *Rosa roxburghii juice* can increase the body’s antioxidant capacity, scavenge reactive oxygen species (ROS), and reduce oxidative damage caused by fluoride and tobacco/alcohol (Jin et al., 2012; Wu et al., 2025).

In terms of blood pressure and lipid regulation, the mechanism by which Drug 3 lowers diastolic blood pressure may be related to improved vascular endothelial function (Lin et al., 2020). Research has shown that the active components in *Gusongbao granules* can promote the release of nitric oxide (NO) by vascular endothelial cells, relax vascular smooth muscle, reduce peripheral vascular resistance, and thereby lower diastolic blood pressure (Chung et al., 2008). The synergistic regulatory effect of Drug 2 combined with sleep improvement and a healthy diet, on systolic blood pressure and blood lipids may involve the regulation of neuroendocrine and metabolic pathways. Good sleep helps maintain normal sympathetic nervous system activity and hormone secretion, reducing the release of angiotensin II and lowering blood pressure (Sayk et al., 2020). A healthy diet (such as increasing dietary fiber intake and reducing saturated fat content) can improve lipid metabolism, lower blood viscosity, and synergistically enhance the lipid-lowering effects of the active components of Drug 2 (He et al., 2024).

This study, which uses an interaction effect model (R^2^ = 0.620 vs. main effect model R^2^ = 0.489), is the first to quantify the synergistic benefits of combined interventions. For example, the combination of Drug 2 and sleep improvement increased the reduction in systolic blood pressure by 41% (*β* = 1.596), whereas the combination of Drug 3 and a healthy diet increased the reduction in diastolic blood pressure by 29% (*β* = −0.848). This “drug-behavior” dynamic matching breaks through the previous ‘single intervention’ research paradigm and provides a causal evidence chain for establishing a comprehensive treatment plan for fluorosis.

Additionally, this study confirmed that smoking/alcohol consumption is not only an independent risk factor but also influences treatment outcomes by modulating drug metabolism pathways. In the Drug 1 group, smoking/drinking increased LDL levels by 37%, whereas Drug 2/Drug 3 maintained their therapeutic advantage in smoking/drinking populations by antagonizing smoking/drinking-induced oxidative stress (e.g., inhibiting NADPH oxidase activity) (Nyquist et al., 2002). This finding fills a critical gap, as previous studies have described only how smoking and drinking exacerbate fluoride toxicity but have not elucidated the regulatory mechanisms underlying their effects on drug response. In the future, smoking/drinking status could serve as a factor in individualized clinical drug selection decisions, such as prioritizing Drug 2/Drug 3 for addicted patients to optimize treatment efficacy.

Using a generalized ordered logit model and hierarchical interaction analysis, we achieved precise quantification of the synergistic effects of “drug-lifestyle” interactions. For example, through *β* value calculations, we found that sleep quality contributes 1.596 units to the blood pressure-lowering effect of Drug 2; a healthy diet enhances the lipid-lowering effect of Drug 3 by 0.848 units. In the future, clinical practice can dynamically adjust treatment plans based on patient behavioral characteristics. For example, for patients with sleep disorders, using Drug 2 instead of Drug 3 can result in an additional 2.27 mmHg reduction in systolic blood pressure (*β* = −2.267, *P* = 0.018).

**FIGURE 5:**
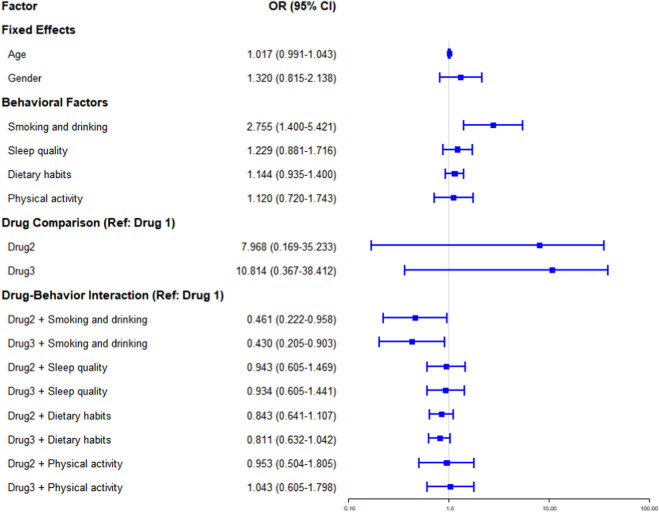
Q-Q plots of propensity scores for Drug 1 And Drug 2 before and after matching.

In conclusion, this study identified smoking and alcohol consumption as significant independent risk factors negatively influencing cardiovascular-metabolic outcomes in skeletal fluorosis patients. Notably, Drug 2 and Drug 3 demonstrated substantially enhanced efficacy among smokers/drinkers, reducing associated risk by 53.9% and 57.0%, respectively, compared to Drug 1. Age exerted heterogeneous effects on blood pressure response, with Drug 3 showing a trend toward superior antihypertensive efficacy. Importantly, smoking and drinking attenuated antihypertensive effects, whereas healthy diets and improved sleep quality synergistically enhanced the efficacy of both Drug 2 and Drug 3. With respect to lipid profiles, age was correlated with changes in LDL and HDL levels. Drug 2 more effectively reduced LDL when coupled with healthy dietary habits, while Drug 3 was associated with a potential reduction in HDL, warranting metabolic caution. Additionally, Drug 2 modestly lowered total cholesterol, and triglyceride levels were primarily influenced by age and smoking/drinking behavior. The proposed interaction framework is illustrated in Figure 5.

Critically, these findings demonstrate that smoking, alcohol consumption, and age significantly modulate drug responses, highlighting the need for personalized treatment strategies that integrate behavioral and demographic factors. This is reinforced by the superior explanatory power of the interaction model over the main-effect model, indicating that individual variation in drug efficacy is more accurately explained by dynamic drug–lifestyle interactions than by drug effects alone. Based on these insights, we propose a novel theoretical framework—PBIT (Personalized Behavioral-Integrated Therapy)—to formalize a paradigm shift from conventional drug-centered prescribing toward an integrated approach that explicitly incorporates patient-specific behavioral contexts. The PBIT framework comprises three core components: (P) personalized profiling using individual behavioral and demographic features; (B) behavioral synergy mapping to identify modifiable lifestyle factors that interact dynamically with pharmacotherapy; and (IT) an integrated therapy platform that combines tailored drug regimens with behavioral monitoring and intervention. By accounting for the complex interplay between pharmacological treatment, lifestyle, and patient factors, the PBIT framework offers a scalable, evidence-based strategy to optimize therapeutic outcomes, mitigate metabolic risks, and advance personalized medicine in fluorosis and other chronic diseases with multifactorial pathophysiology.

This study has several limitations that warrant attention. The 3-month intervention period may be insufficient to capture long-term changes in skeletal fluorosis staging, as the progression of this chronic condition typically unfolds over extended durations. Furthermore, the lack of genetic profiling and pharmacokinetic monitoring restricts the ability to differentiate between pharmacodynamic and metabolic influences on therapeutic efficacy. Future investigations should extend follow-up durations with more frequent observation nodes and incorporate multi-omics approaches—such as genomics and metabolomics—to holistically examine the interactions among genetic backgrounds, drug metabolism, and clinical outcomes. For instance, future studies could leverage metabolomic technologies to elucidate the mechanisms through which Drug 2 and Drug 3 sustain efficacy despite tobacco and alcohol exposure, aligning with our proposed “PBIT framework”paradigm—a core theoretical contribution of this research that emphasizes optimizing personalized interventions through synergistic drug-lifestyle pairing. Such approaches would not only advance mechanistic understanding of TCM efficacy but also promote precision medicine in skeletal fluorosis management.

## Data Availability

The original contributions presented in the study are included in the article/Supplementary Material, further inquiries can be directed to the corresponding authors.

## References

[B1] Amini H. Taghavi Shahri S. M. Amini M. Ramezani Mehrian M. Mokhayeri Y. Yunesian M. (2011). Drinking water fluoride and blood pressure? An environmental study. Biol. Trace Elem. Res. 144 (1), 157–163. 10.1007/s12011-011-9054-5 21484404

[B2] Angwa L. M. Nyadanu S. D. Kanyugo A. M. Adampah T. Pereira G. (2023). Fluoride-induced apoptosis in non-skeletal tissues of experimental animals: a systematic review and meta-analysis. Heliyon 9 (8), e18646. 10.1016/j.heliyon.2023.e18646 37560699 PMC10407679

[B3] Bai B. B. Xie X. W. Li D. P. (2018). Current status of epidemiological research on osteoporosis in China in recent five years. Chin. J. Osteoporos. 24 (02), 253–258. 10.3969/j.issn.1006-7108.2018.02.024

[B4] Barbier O. Arreola-Mendoza L. Del Razo L. M. (2010). Molecular mechanisms of fluoride toxicity. Chem. Biol. Interact. 188 (2), 319–333. 10.1016/j.cbi.2010.07.011 20650267

[B5] Chauhan S. S. Ojha S. Mahmood A. (2013). Effects of fluoride and ethanol administration on lipid peroxidation systems in rat brain. Indian J. Exp. Biol. 51 (3), 249–255. 23678546

[B6] Chinese Pharmacopoeia Commission (2020). Pharmacopoeia of the people’s Republic of China: volume I. Beijing: China Medical Science Press, 338–339.

[B7] Chung B. H. Kim J. D. Kim C. K. Won M. H. (2008). Icariin stimulates angiogenesis by activating the MEK/ERK- and PI3K/Akt/eNOS-dependent signal pathways in human endothelial cells. Biochem. Biophys. Res. Commun. 376 (2), 404–408. 10.1016/j.bbrc.2008.09.001 18789310

[B8] Fu Y. Y. Liu J. M. Lu X. L. (2020). Research progress on main active components and pharmacological effects of Rosa roxburghii tratt. Sci. Technol. Food Industry 41 (13), 328–335+342. 10.13386/j.issn1002-0306.2020.13.052

[B9] Gao X. M. (2007). Chinese materia medica. Beijing: China Press of Traditional Chinese Medicine, 456–457.

[B10] He X. Wang J. Li M. Hao D. Yang Y. Zhang C. (2014). Eucommia ulmoides oliv.: ethnopharmacology, phytochemistry and pharmacology of an important traditional Chinese medicine. J. Ethnopharmacol. 151 (1), 78–92. 10.1016/j.jep.2013.11.023 24296089

[B11] He D. Wang H. Zhang M. Yang J. Yuan M. Gong T. (2024). Dietary fiber improves lipid metabolism through changes in gut microbiota and their metabolites in high-fat diet fed rats. J. Biol. Regul. Homeost. Agents 38 (2), 1409–1420. 10.23812/j.biol.regul.homeost.agents.20243802.111

[B12] Jin M. Kumar A. Kumar S. (2012). Ethanol-mediated regulation of cytochrome P450 2A6 expression in monocytes: role of oxidative stress-mediated PKC/MEK/Nrf2 pathway. PLoS One 7 (4), e35505. 10.1371/journal.pone.0035505 22530035 PMC3329463

[B13] Johnson J. T. Hussain M. A. Cherian K. E. Kapoor N. Paul T. V. (2022). Chronic alcohol consumption and its impact on bone and metabolic Health–a narrative review. Indian J. Endocrinol. Metab. 26 (3), 206–212. 10.4103/ijem.ijem_26_22 36248052 PMC9555370

[B14] Johnston N. R. Strobel S. A. (2020). Principles of fluoride toxicity and the cellular response: a review. Arch. Toxicol. 94 (4), 1051–1069. 10.1007/s00204-020-02687-5 32152649 PMC7230026

[B15] Khudhur Z. O. Smail S. W. Awla H. K. Ahmed G. B. Khdhir Y. O. Amin K. (2025). The effects of heavy smoking on oxidative stress, inflammatory biomarkers, vascular dysfunction, and hematological indices. Sci. Rep. 15 (1), 18251. 10.1038/s41598-025-03075-8 40415015 PMC12104322

[B16] Kroon L. A. (2007). Drug interactions with smoking. Am. J. Health Syst. Pharm. 64 (18), 1917–1921. 10.2146/ajhp060414 17823102

[B17] Kwak S. C. Lee C. Kim J.-Y. Oh H. M. So H. S. Lee M. S. (2013). Chlorogenic acid inhibits osteoclast differentiation and bone resorption by down-regulation of receptor activator of nuclear factor kappa-B ligand-induced nuclear factor of activated T cells c1 expression. Biol. Pharm. Bull. 36 (11), 1779–1786. 10.1248/bpb.b13-00430 23985829

[B18] Li B. Ren T. Y. (2022). Research progress on main active components and pharmacological effect of Rosa roxburghii tratt. Guizhou Agric. Sci. 50 (11), 84–92. 10.3969/j.issn.1001-3601.2022.11.013

[B19] Li Y. Berenji G. R. Shaba W. F. Tafti B. Yevdayev E. Dadparvar S. (2012). Association of vascular fluoride uptake with vascular calcification and coronary artery disease. Nucl. Med. Commun. 33 (1), 14–20. 10.1097/MNM.0b013e32834c187e 21946616

[B20] Li J. Liu S. Cao G. Sun Y. Chen W. Dong F. (2018). Nicotine induces endothelial dysfunction and promotes atherosclerosis *via* GTPCH 1. J. Cell Mol. Med. 22 (11), 5406–5417. 10.1111/jcmm.13812 30091833 PMC6201367

[B21] Li M. Zhao Y. Tian X. Liu P. Xie J. Dong N. (2021). Fluoride exposure and blood pressure: a systematic review and meta-analysis. Biol. Trace Elem. Res. 199 (3), 925–934. 10.1007/s12011-020-02232-6 32602052

[B22] Lin X. Han T. Fan Y. Wu S. Wang F. Wang C. (2020). Quercetin improves vascular endothelial function through promotion of autophagy in hypertensive rats. Life Sci. 258, 118106. 10.1016/j.lfs.2020.118106 32682916

[B23] Liu X. Hao W. Qin Y. Decker Y. Wang X. Burkart M. (2015). Long-term treatment with Ginkgo biloba extract EGb 761 improves symptoms and pathology in a transgenic mouse model of Alzheimer's disease. Brain, Behav. Immun. 46, 121–131. 10.1016/j.bbi.2015.01.011 25637484

[B24] Loffredo L. Zicari A. M. Occasi F. Perri L. Carnevale R. Angelico F. (2018). Role of NADPH oxidase-2 and oxidative stress in children exposed to passive smoking. Thorax 73 (10), 986–988. 10.1136/thoraxjnl-2017-211293 29449441

[B25] Lu J. H. Li Z. Y. Du G. Q. Zhang J. Wang Y. P. Shi J. Y. (2023). Systematic review and meta-analysis of gusongbao preparation for primary osteoporosis. China J. Chin. Mater. Med. 48(11). 3086–3096. 10.19540/j.cnki.cjcmm.20230130.501 37381967

[B26] Meena L. Gupta R. (2021). Skeletal fluorosis. N. Engl. J. Med. 385 (16), 1510. 10.1056/NEJMicm2103503 34623787

[B27] Nyquist F. Düppe H. Obrant K. J. Bondeson L. Nordsletten L. (2002). Effects of alcohol on bone mineral and mechanical properties of bone in male rats. Alcohol Alcohol 37 (1), 21–24. 10.1093/alcalc/37.1.21 11825852

[B28] Pereira H. A. Dionizio A. S. Fernandes M. S. Araujo T. T. Cestari T. M. Buzalaf C. P. (2016). Fluoride intensifies hypercaloric diet-induced ER oxidative stress and alters lipid metabolism. PLoS One 11 (6), e0158121. 10.1371/journal.pone.0158121 27336443 PMC4919043

[B29] Pramanik S. Saha D. (2017). The genetic influence in fluorosis. Environ. Toxicol. Pharmacol. 56, 157–162. 10.1016/j.etap.2017.09.008 28938149

[B30] Rejnmark L. Vestergaard P. Heickendorff L. Mosekilde L. (2010). Simvastatin does not affect vitamin d status, but low vitamin d levels are associated with dyslipidemia: results from a randomised, controlled trial. Int. J. Endocrinol. 2010 (1), 957174. 10.1155/2010/957174 20016680 PMC2778175

[B31] Sayk F. Twesten C. Adametz I. Franzen K. Vonthein R. Dodt C. (2020). Angiotensin II-mediated nondipping during sleep in healthy humans: effects on baroreflex function at subsequent daytime. Am. J. Physiol. Regul. Integr. Comp. Physiol. 318 (4), R813–R821. 10.1152/ajpregu.00355.2019 32130025

[B32] Sheikh A. Collaborators GBDA (2018). Alcohol use and burden for 195 countries and territories, 1990–2016: a systematic analysis for the global burden of disease study 2016. Lancet 392 (10152), 1015–1035. 10.1016/S0140-6736(18)31310-2 30146330 PMC6148333

[B33] Shen J. Zhang S. Zhang J. Wei X. Wang Z. Han B. (2024). Osteogenic mechanism of chlorogenic acid and its application in clinical practice. Front. Pharmacol. 15, 1396354. 10.3389/fphar.2024.1396354 38873428 PMC11169668

[B34] Spence L. A. Weaver C. M. (2013). Calcium intake, vascular calcification, and vascular disease. Nutr. Rev. 71 (1), 15–22. 10.1111/nure.12002 23282248

[B35] Sun L. Gao Y. Zhang W. Liu H. Sun D. (2014). Effect of high fluoride and high fat on serum lipid levels and oxidative stress in rabbits. Environ. Toxicol. Pharmacol. 38 (3), 1000–1006. 10.1016/j.etap.2014.10.010 25461561

[B36] Sun D. Gao Y. Liu H. (2019). Achievements and prospects of endemic disease prevention and control in China in past 70 years. Chin. J. Public Health 35 (7), 793–796. 10.11847/zgggws1124576

[B37] Sun D. Liu H. Yu J. (2023). Current main problems and countermeasures for prevention and control of endemic diseases in China. Chin. J. Epidemiol. 42 (1), 1–3. 10.3760/cma.j.cn231583-20221224-00412

[B38] Varol E. Varol S. (2013). Comment on: does fluoride toxicity cause hyperlipidaemia and hyperglycaemia in patients with endemic fluorosis? J. Sci. Food Agric. 93 (2), 427. 10.1002/jsfa.5938 23239388

[B39] Wölfl J. Dagher M. C. Fuchs A. Geiszt M. Ligeti E. (1996). *In vitro* activation of the NADPH oxidase by fluoride: possible involvement of a factor activating GTP hydrolysis on rac (Rac‐GAP). Eur. J. Biochem. 239 (2), 369–375. 10.1111/j.1432-1033.1996.0369u.x 8706742

[B40] Wu H. Xu C. Yan Y. Peng M. Deng T. Yang X. (2025). Antioxidant activity and whitening effect of vitamin C and superoxide dismutase from Rosa roxburghii tratt. Food Qual. Saf. 9, fyae046. 10.1093/fqsafe/fyae046

[B41] Xia M. F. Chen L. Y. Wu L. Ma H. Li X. M. Li Q. (2021). Sarcopenia, sarcopenic overweight/obesity and risk of cardiovascular disease and cardiac arrhythmia: a cross-sectional study. Clin. Nutr. 40 (2), 571–580. 10.1016/j.clnu.2020.06.003 32593523

[B42] Xu R. Xu R. (1997). Electrocardiogram analysis of patients with skeletal fluorosis. Fluoride 30 (1), 16–18.

[B43] Yang C. Wang Y. Xu H. (2017). Treatment and prevention of skeletal fluorosis. Biomed. Environ. Sci. 30 (2), 147–149. 10.3967/bes2017.020 28292354

[B44] Yang S. Huang X.-Y. Zhou N. Wu Q. Liu J. Shi J.-S. (2022). RNA-seq analysis of protection against chronic alcohol liver injury by Rosa roxburghii fruit juice (Cili) in mice. Nutrients 14 (9), 1974. 10.3390/nu14091974 35565941 PMC9104053

[B45] Yang W. Lu C. Chu F. Bu K. Ma H. Wang Q. (2024). Fluoride-induced hypertension by regulating RhoA/ROCK pathway and phenotypic transformation of vascular smooth muscle cells: *in vitro* and *in vivo* evidence. Ecol. Toxicol. Environ. Saf. 281, 116681. 10.1016/j.ecoenv.2024.116681 38964063

[B46] Yao Z. H. Qin Z. F. Cheng H. Wu X. M. Dai Y. Wang X. L. (2017). Simultaneous quantification of multiple representative components in the xian-ling-gu-bao capsule by ultra-performance liquid chromatography coupled with quadrupole time-of-flight tandem mass spectrometry. Molecules 22 (6), 927. 10.3390/molecules22060927 28574448 PMC6152775

[B47] Zhang D. W. Cheng Y. Wang N. L. Zhang J. C. Yang M. S. Yao X. S. (2008). Effects of total flavonoids and flavonol glycosides from Epimedium koreanum Nakai on the proliferation and differentiation of primary osteoblasts. Phytomedicine 15 (1-2), 55–61. 10.1016/j.phymed.2007.04.002 17482445

[B48] Zhang N. D. Han T. Huang B. K. Rahman K. Jiang Y. P. Xu H. T. (2016). Traditional Chinese medicine formulas for the treatment of osteoporosis: implication for antiosteoporotic drug discovery. J. Ethnopharmacol. 189, 61–80. 10.1016/j.jep.2016.05.025 27180315

[B49] Zhang P. F. Liao L. J. Deng Z. (2017). Research progress on pharmacological effects and clinical application of Ginkgo biloba extract. Liaoning J. Traditional Chin. Med. 44(02): 426–429. 10.13192/j.issn.1000-1719.2017.02.069

[B50] Zhao L. Sun Y. Yu G. (2011). The sole standard for evaluating the effectiveness of endemic fluorosis prevention and control: interpretation of the control standard for endemic fluorosis areas (GB17017- 2010). China Health Stand Manag. 2 (2), 37–40.

[B51] Zhou Q. H. Zhang D. C. (1988). Electrocardiogram analysis of 271 dental fluorosis cases. Chin. J. Endem. 5, 296–297.

