# Supplementary material for: Exploring the therapeutic synergy of drug-lifestyle interventions in fluorosis: a randomized trial on cardiovascular metabolic outcomes from the China fluorosis cohort (CFC)
Source: Front Pharmacol. 2026 Mar 31;17:1737666. doi: 10.3389/fphar.2026.1737666 (PMC13076280; doi:10.3389/fphar.2026.1737666)
Supplement: Supplementary file 1 [file Table1.doc]

Appendix Table A1. Assessment Indicators and Scoring Criteria for Behaviors*

| **Category** | **Item** | **Options** | **Score** |
| --- | --- | --- | --- |
| Smoking and drinking(Scored 0–11 points) | Smoking status | non-smoker | 1 |
|  |  | quit smoking | 2 |
|  |  | current smoker (cumulatively over 100 cigarettes) | 3 |
|  | Passive smoking | no | 0 |
|  |  | yes | 1 |
|  | Drinking frequency in the past year | never or almost never | 0 |
|  |  | only occasionally on special occasions (e.g., festivals) | 1 |
|  |  | only in specific months of the year (e.g., busy farming seasons or summer) | 2 |
|  |  | monthly drinking regardless of season, less than once a week | 3 |
|  |  | at least once a week year-round | 4 |
|  | Excessive drinking leading to inability to work | no | 0 |
|  |  | yes | 1 |
|  | Depressed mood after drinking | no | 0 |
|  |  | yes | 1 |
|  | Alcohol addiction | no | 0 |
|  |  | yes | 1 |
|  | Body shaking after stopping drinking | no | 0 |
|  |  | yes | 1 |
| Sleep quality(Scored 0–7 points) | Taking more than 30 minutes to fall asleep on at least 3 days per week | no | 0 |
|  |  | yes | 1 |
|  | Waking up early and failing to fall back asleep on at least 3 days per week | no | 0 |
|  |  | yes | 1 |
|  | Taking sleeping pills (Western or Chinese medicine) at least 1 day per week | no | 0 |
|  |  | yes | 1 |
|  | Difficulty staying alert during work, meals, or conversations due to poor sleep on at least 3 days per week | no | 0 |
|  |  | yes | 1 |
|  | Napping habit | year-round | 0 |
|  |  | yes, but usually only in summer | 1 |
|  |  | no | 2 |
|  | Average daily sleep duration | 6–8 hours | 0 |
|  |  | less than or more than 6–8 hours | 1 |
| Dietary habits(Scored 3–16 points) | Grain intake | <1 time/week | 1 |
|  |  | <3 times/week | 2 |
|  |  | 3–6 times/week | 3 |
|  |  | ≥1 time/day | 4 |
|  | Meat and egg intake | <1 time/week | 1 |
|  |  | <3 times/week | 2 |
|  |  | 3–6 times/week | 3 |
|  |  | ≥1 time/day | 4 |
|  | Fresh vegetable and fruit intake | <1 time/week | 1 |
|  |  | <3 times/week | 2 |
|  |  | 3–6 times/week | 3 |
|  |  | ≥1 time/day | 4 |
|  | Dairy and product intake | never | 0 |
|  |  | <1 time/week | 1 |
|  |  | <3 times/week | 2 |
|  |  | 3–6 times/week | 3 |
|  |  | ≥1 time/day | 4 |
| Physical activity(Scored 0–3 points) | Daily extra exercise time (excluding labor) | none | 0 |
|  |  | <30 minutes | 1 |
|  |  | 30 minutes–1 hour | 2 |
|  |  | >1 hour | 3 |

*The assessment of sleep quality refers to some dimensions of the Pittsburgh Sleep Quality Index (PSQI). The sections on smoking and drinking refer to some relevant dimensions of the Alcohol Use Disorders Identification Test (AUDIT) and the Global Adult Tobacco Survey (GATS). The scoring of dietary habits is based on research related to healthy diets and dietary guidelines.
