# Supplementary material for: Exploring the therapeutic synergy of drug-lifestyle interventions in fluorosis: a randomized trial on cardiovascular metabolic outcomes from the China fluorosis cohort (CFC)
Source: Front Pharmacol. 2026 Mar 31;17:1737666. doi: 10.3389/fphar.2026.1737666 (PMC13076280; doi:10.3389/fphar.2026.1737666)
Supplement: Supplementary file 2 [file Table2.doc]

Appendix Table A2. Generalized Linear Mixed Model Results for blood pressure and lipids, *β* (SE).

*Note: β (regression coefficient); SE (standard error); DBP (Diastolic Blood Pressure); SBP (Systolic Blood Pressure); HDL (High-Density Lipoprotein); LDL (Low-Density Lipoprotein); TC (Total Cholesterol); TG (Triglycerides).*
